# The expression profile and clinic significance of the SIX family in non-small cell lung cancer

**DOI:** 10.1186/s13045-016-0339-1

**Published:** 2016-11-08

**Authors:** Qian Liu, Anping Li, Yijun Tian, Yu Liu, Tengfei Li, Cuntai Zhang, Jennifer D. Wu, Xinwei Han, Kongming Wu

**Affiliations:** 1 Department of Oncology, Tongji Hospital of Tongji Medical College, Huazhong University of Science and Technology, Wuhan, 430030 China; 2 Department of Interventional Radiology, The First Affiliated Hospital of Zhengzhou University, Zhengzhou, 450052 China; 3 Department of Geriatric, Tongji Hospital of Tongji Medical College, Huazhong University of Science and Technology, Wuhan, 430030 China; 4 Department of Microbiology and Immunology, Hollings Cancer Center, Medical University of South Carolina, Charleston, SC USA

**Keywords:** SIX family genes, Lung cancer, Tumor marker, Prognosis, Meta-analysis

## Abstract

**Background:**

The SIX family homeobox genes have been demonstrated to be involved in the tumor initiation and progression, but their clinicopathological features and prognostic values in non-small cell lung cancer (NSCLC) have not been well defined. We analyzed relevant datasets and performed a systemic review and a meta-analysis to assess the profile of SIX family members in NSCLC and evaluate their importance as biomarkers for diagnosis and prediction of NSCLC.

**Methods:**

This meta-analysis included 17 studies with 2358 patients. Hazard ratio (HR) and 95 % confidence interval (CI) were calculated to represent the prognosis of NSCLC with expression of the SIX family genes. Heterogeneity of the ORs and HRs was assessed and quantified using the Cochrane *Q* and *I* ^2^ test. Begg’s rank correlation method and Egger’s weighted regression method were used to screen for potential publication bias. Bar graphs of representative datasets were plotted to show the correlation between the SIX expression and clinicopathological features of NSCLC. Kaplan-Meier survival curves were used to validate our prognostic analysis by pooled HR.

**Results:**

The systematic meta-analysis unveiled that the higher expressions of SIX1-5 were associated with the greater possibility of the tumorigenesis. SIX4 and SIX6 were linked to the lymph node metastasis (LNM). SIX2, SIX3, and SIX4 were correlated with higher TNM stages. Furthermore, the elevated expressions of SIX2, SIX4, and SIX6 predicted poor overall survival (OS) in NSCLC (SIX2: HR = 1.14, 95 % CI, 1.00–1.31; SIX4: HR = 1.39, 95 % CI, 1.16–1.66; SIX6: HR = 1.18, 95 % CI, 1.00–1.38) and poor relapse-free survival (RFS) in lung adenocarcinoma (ADC) (SIX2: HR = 1.42, 95 % CI, 1.14–1.77; SIX4: HR = 1.52, 95 % CI, 1.09–2.11; SIX6: HR = 1.25, 95 % CI, 1.01–1.56).

**Conclusions:**

Our report demonstrated that the SIX family members play distinct roles in the tumorigenesis of NSCLC and can be potential biomarkers in predicting prognosis of NSCLC patients.

**Electronic supplementary material:**

The online version of this article (doi:10.1186/s13045-016-0339-1) contains supplementary material, which is available to authorized users.

## Backgrounds

The members of Retinal Determination Gene Network (RDGN), mainly including DACH, SIX, and EYA, elucidated as a cascaded signal pathway regulating precursor cell proliferation, differentiation, and survival during mammalian organogenesis in a cell context-dependent manner [1–4]. Specifically, decreased EYA1 and SIX1 expression during the late pseudoglandular stage involving epithelial branching and distal airway maturation led to the pulmonary hypoplasia (PH) [5]. Transgenic mouse model demonstrated that deletion of either Six1 or Eya1 genes leads to a defect of mesenchymal cell development and remodeling of the distal lung septae and arteries [6]. It has been proved that EYA1 and SIX1 played pivotal roles during lung morphogenesis [7]. Molecular mechanism study indicated that in RDGN signaling, SIX functioned as a DNA-binding transcriptional factor, while EYA and DACH served as co-activator and co-repressor of SIX family, respectively, to control gene activation or repression [4]. However, it was proposed that EYA did not interact with SIX3 as a co-activator [8].

The SIX family consists of six members divided into three subfamilies, namely SIX1/SIX2 (*So*), SIX3/SIX6 (*Optix*), and SIX4/SIX5 (*Dsix4*) [9, 10]. The SIX family members are characterized by two evolutionarily conserved domains. The SIX domain (SD) is involved in protein–protein interactions, while homeobox nucleic acid recognition domain (HD) is related to DNA binding [11, 12]. SIX family binds to DNA at a core consensus sequence of TAAT, whereas SIX3 binds to an additional TGATAC sequence [13]. It has been reported that SIX family modulates a series of key genes, namely cyclin A1, cyclin D1, and c-myc. Sufficient evidences have revealed that aberrant expressions of the SIX genes gave rise to tumorigenesis, tumor progression, and metastasis by promoting proliferation, angiogenesis, migration, and apoptosis [14].

The SIX family was reported to be related with non-small cell lung cancer (NSCLC) recently. Study from Xia Y et al. indicated that SIX1 promoted the invasion and proliferation of NSCLC [15]. And Mimae T et al. showed that upregulation of SIX1 in both messenger RNA (mRNA) and protein expressions led to lung adenocarcinoma (ADC) invasion by inducing epithelial-mesenchymal transition (EMT) [16]. Moreover, Zhao Y et al. proposed that SIX6 combined with other co-factors were considered as promoters to the development of lung squamous cell carcinoma (SQC) [17]. However, another critical member of SIX family, SIX3, was reported to act as a repressor in ADC cell proliferation and migration. More importantly, SIX3 could remarkably improve overall survival (OS) and progression-free survival (PFS) in early stage ADC patients [18]. To comprehensively explore the effect of different SIX family members in NSCLC, we analyzed relevant datasets and performed a systemic review and a meta-analysis to assess the profile of SIX family members in NSCLC and evaluate their importance as biomarkers for diagnosis and prediction of NSCLC.

## Methods

### Literature retrieval

The relevant literatures were obtained from following databases PubMed, Embase, and Cochrane library published up to October 1, 2015 using the search terms NSCLC (non-small-cell lung cancer, non-small cell lung cancer), SIX1 (SIX1 protein, human), SIX2 (SIX2 protein, human), SIX3 (Sine oculis homeobox homolog 3 protein), SIX4 (SIX4 protein, human), SIX5 (SIX5 protein,human), and SIX6 (SIX6 protein, human). The reference list including retrieved articles was reviewed to discover possible associated publications.

### Inclusion criteria

Randomized controlled studies (RCTs) or case-control or cohort studies were selected in this meta-analysis to evaluate the correlation between the SIX family expression and NSCLC clinicopathological features and prognosis. The following criteria were strictly observed: (a) patients recruited into the study were pathologically diagnosed as NSCLC; (b) the expressions of the SIX family genes were extracted from normalized microarray within primary NSCLC tumor, and median expression was used as cut-off value. Detailed information for genechips and platforms was in Table 1; (c) hazard ratio (HR) and 95 % CI were available or statistically extracted from relevant literatures [19]. As for reports with the same population, the most recent or complete report was chosen.

**Table 1:** Characteristics of studies included for meta-analysis. Cut-off value: median expression

First author	Year	Duration (months)	Histology	Stage	Patient number	Quality score	Detection	Platform
Okayama H [41]	2011	120	ADC	I–II	226	9	Microarray	Affymetrix Hgu133plus2.0
Hou J [42]	2010	130	NSCLC	NR	91	9	Microarray	AffymetrixHgu133plus2.0
Tang H [43]	2013	120	NSCLC	I–III	176	9	Microarray	IlluminaHumanWG-6v3.0
Zhu CQ [44]	2010	108	NSCLC	IB–II	133	9	Microarray	AffymetrixHgu133a
Raponi M [45]	2006	144	SQC	I–III	129	8	Microarray	AffymetrixHgu133plus2.0
Bild AH [46]	2006	88	NSCLC	NR	111	8	Microarray	AffymetrixHgu133plus2.0
Rousseaux S [47]	2013	256	NSCLC	I–IV	293	9	Microarray	AffymetrixHgu133plus2.0
Tomida S [48]	2009	109.8	ADC	I–III	117	9	Microarray	AgilentWholeHumanGenomeMicroarray 4x44K G4112F
Botling J [49]	2013	120	NSCLC	I–IV	196	8	Microarray	AffymetrixHgu133plus2.0
Shedden K [50]	2008	204	ADC	I–IV	333	9	Microarray	AffymetrixHgu133a
Beer DG [51]	2002	110.6	ADC	I–III	86	8	Microarray	AffymetrixHumanFullLength HuGeneFL
Landi MT [52]	2008	NR	ADC	I–IV	74	9	Microarray	AffymetrixHgu133a
Lu T [53]	2010	NR	ADC	I–IV	60	8	Microarray	AffymetrixHgu133plus2.0
Lee ES [54]	2008	120	NSCLC	I–III	138	8	Microarray	AffymetrixHgu133plus2.0
Kuner R [55]	2009	NR	NSCLC	NR	58	8	Microarray	AffymetrixHgu133plus2.0
Selamat SA [56]	2012	NR	ADC	I–III	58	8	Microarray	IlluminaHumanWG-6v3.0 expression
Meyerson M [57]	2015	NR	SQC	I–IV	135	8	Microarray	AffymetrixHgu133a

*NR* not reporting, *ADC* lung adenocarcinoma, *SQC* lung squamous cell carcinoma

### Data extraction

All data were presented in Table 1 with the following information: first author’s last name, publication year, duration month, histological type, tumor stage, number of cases and controls, and detection methods and platforms for the SIX family. The levels of gene expressions and NSCLC survival data were obtained from Oncomine and ArrayExpress. OS and relapse-free survival (RFS) were calculated by Cox proportional HRs and 95 % CIs.

The Newcastle-Ottawa Quality Assessment Scale (NOS) was applied to evaluate the quality of these observational studies. Data from included studies were extracted and summarized independently by two reviewers, and disagreements were settled by discussion.

### Statistical analysis

Meta-Analysis of Observational Studies served as guidelines applied for statistical analysis [20]. HRs and 95 % CIs were calculated to represent the prognosis of NSCLC with expression of the SIX family genes. Clinicopathological parameters included histological type, lymph node metastasis (LNM), and TNM stage. Heterogeneity of the ORs and HRs was assessed and quantified using Cochrane *Q* and *I* ^2^ test. Random-effect model was employed if there was heterogeneity between studies (*p* < 0.05 or *I* ^2^ > 50 %). Otherwise, fixed-effect model was applied. Begg’s rank correlation method and Egger’s weighted regression method were used to screen for potential publication bias. All *p* values were two tailed, and all analyses were accomplished using STATA software package (version 13.0) (Stata Corp LP, College Station, TX, USA). We selected the representative datasets, GSE19188, GSE19804, and GSE32863 to analyze the significance of SIX expression in clinicopathological features of NSCLC. The bar graphs were printed using GraphPad Prism 5.0 software. Unpaired *t* test was used to determine differences between groups.

### Kaplan-Meier plotter

Kaplan-Meier survival curves with hazard ratio and logrank *p* value were calculated and plotted with the analysis tool which can be accessed online at http://kmplot.com/analysis/ [21]. The background database, downloaded from GEO (Affymetrix microarrays only), EGA, and TCGA, offers gene expression data, relapse-free, and overall survival information. The software is capable to assess the effect of 54,675 genes on survival using 10,188 cancer samples, among which includes 2437 cases of lung cancer. We analyzed the survival outcomes of NSCLC in different expression levels of SIX2, SIX4, and SIX6. The Kaplan-Meier survival curves were downloaded from the website and resized in Adobe Illustrator CS5.

## Results

### Search results

**Fig. 1:**
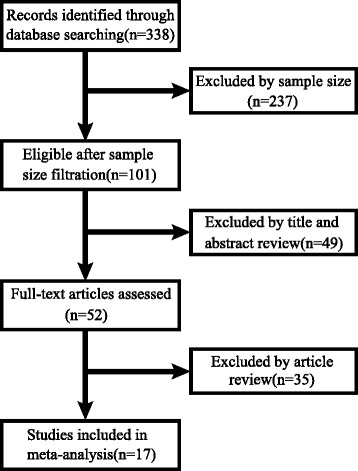
Flow diagram of literature retrieval process

The flow diagram reflecting the selection process for inclusive studies is illustrated in Fig. 1. Three hundred thirty-eight studies were screened out depending on mentioned search strategy. According to requirement for sample size (*n* ≥ 50), 237 studies were excluded. After selecting by assessing article titles, abstracts, and full-texts, 17 studies with 2358 cases were applied to this analysis. These studies primarily focused on the causality between the SIX family expression and the NSCLC progression and prognosis. The clinicopathological parameters such as histological type, LNM, and TNM stage were available in every study. The information of relevant literatures was listed in the Table 1. TNM stages I and II were defined as low stage, III and IV as high stage.

### The expressions of SIX family genes elevated in NSCLC tissues

**Fig. 2:**
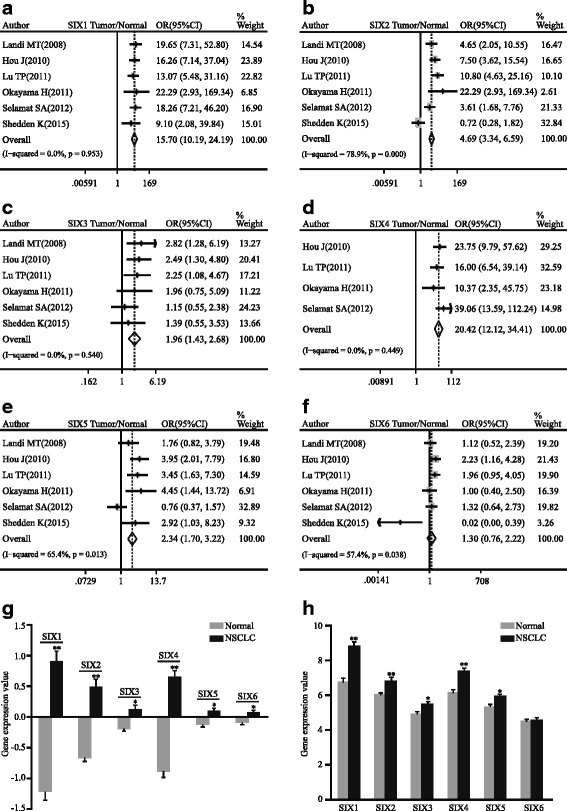
Forest plot of odds ratio (OR). *CI* confidence interval. Relative mRNA level of SIX1 (**a**), SIX2 (**b**), SIX3 (**c**), SIX4 (**d**), SIX5 (**e**), and SIX6 (**f**) in NSCLC compared with normal lung tissues. Bar graph representation of relative SIX family mRNA level of NSCLC compared with normal lung tissues in GSE19188 (**g**) and GSE19804 (**h**). *p < 0.05, **p < 0.0001

**Fig. 3:**
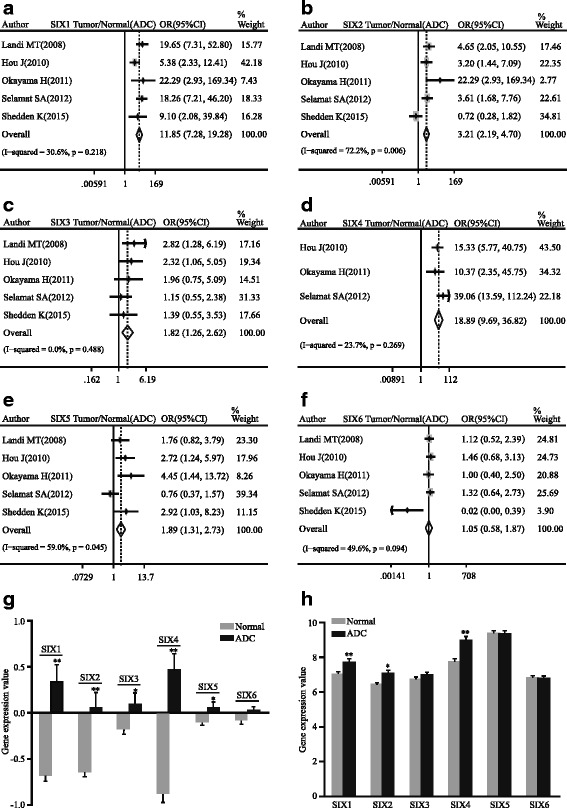
Forest plot of odds ratio (OR). *CI* confidence interval. Relative mRNA level of SIX1 (**a**), SIX2 (**b**), SIX3 (**c**), SIX4 (**d**), SIX5 (**e**), and SIX6 (**f**) in ADC compared with normal lung tissues. Bar graph representation of relative SIX family mRNA level of ADC compared with normal lung tissues in GSE19188 (**g**) and GSE32863 (**h**). *p < 0.05, **p < 0.0001

The SIX family genes, especially SIX1, SIX2, SIX3, SIX4, and SIX5, manifested significantly higher expression level in NSCLC tissues than normal lung tissues (SIX1: pooled OR = 15.70, 95 % CI, 10.19–24.19, *p* = 0.953, and *I* ^2^ = 0.0 %; SIX2: pooled OR = 4.69, 95 % CI, 3.34–6.59, *p* = 0.000, and *I* ^2^ = 78.9 %; SIX3: pooled OR = 1.96, 95 % CI, 1.43–2.68, *p* = 0.540, and *I* ^2^ = 0.0 %; SIX4: pooled OR = 20.42, 95 % CI, 12.12–34.41, *p* = 0.449, and *I* ^2^ = 0.0 %; SIX5: pooled OR = 2.34, 95 % CI, 1.70–3.22, *p* = 0.013, and *I* ^2^ = 65.4 %; Fig. 2a–e). Nevertheless, SIX6 showed a considerable trend toward significance (pooled OR = 1.30, 95 % CI, 0.76–2.22, *p* = 0.038, and *I* ^2^ = 57.4 %; Fig. 2f). Subgroup analysis of ADC was also proven to have the similar trend (SIX1: pooled OR = 11.85, 95 % CI, 7.28–19.28, *p* = 0.218, and *I* ^2^ = 30.6 %; SIX2: pooled OR = 3.21, 95 % CI, 2.19–4.70, *p* = 0.006, and *I* ^2^ = 72.2 %; SIX3: pooled OR = 1.82, 95 % CI, 1.26–2.62, *p* = 0.488, and *I* ^2^ = 0.0 %; SIX4: pooled OR = 18.89, 95 % CI, 9.69–36.82, *p* = 0.269, and *I* ^2^ = 23.7 %; SIX5: pooled OR = 1.89, 95 % CI, 1.31–2.73, *p* = 0.045, and *I* ^2^ = 59.0 %; Fig. 3a–e). SIX6 indicated uncertain significance (pooled OR = 1.05, 95 % CI, 0.58–1.87, *p* = 0.094, and *I* ^2^ = 49.6 %; Fig. 3f). Expression of SIX family between normal and NSCLC tissues in the representative dataset GSE19188 showed statistical difference (SIX1: *p* < 0.0001; SIX2: *p* < 0.0001; SIX3: *p* = 0.004; SIX4: *p* < 0.0001; SIX5: *p* = 0.0027; SIX6: *p* = 0.0025; Fig. 2g), while the similar tendency was shown in another representative dataset GSE19804 except SIX6 (SIX1: *p* < 0.0001; SIX2: *p* < 0.0001; SIX3: *p* = 0.0146; SIX4: *p* < 0.0001; SIX5: *p* = 0.0098; SIX6: *p* = 0.0615; Fig. 2h). In ADC patients, the expression levels of SIX1-5 were markedly higher than normal in GSE19188 (SIX1: *p* < 0.0001; SIX2: *p* < 0.0001; SIX3: *p* = 0.0129; SIX4: *p* < 0.0001; SIX5: *p* = 0.0193; SIX6: *p* = 0.077; Fig. 3g). In GSE32863, only SIX1, SIX2, and SIX4 indicated significant differences between ADC and normal tissues (SIX1: *p* < 0.0001; SIX2: *p* = 0.0002; SIX3: *p* = 0.1021; SIX4: *p* < 0.0001; SIX5: *p* = 0.6497; SIX6: *p* = 0.5539; Fig. 3h).

### The SIX family gene expressions correlated with TNM stage of NSCLC

**Fig. 4:**
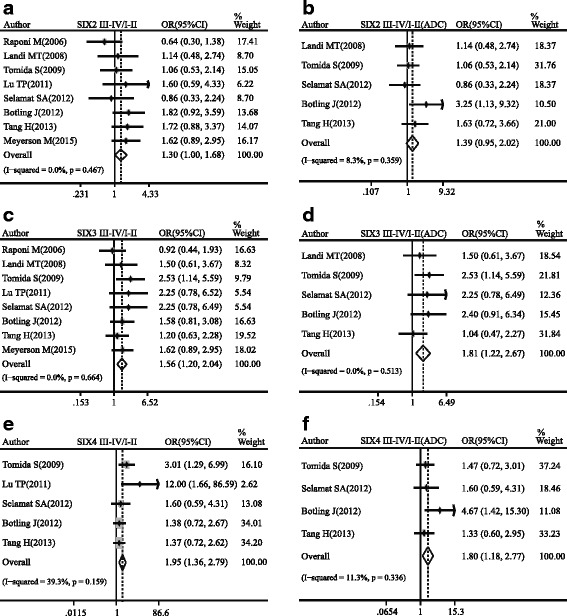
Forest plot of odds ratio (OR). *CI* confidence interval. Relative mRNA expression of SIX2 (**a**), SIX3 (**c**), and SIX4 (**e**) between III–IV and I–II patients in NSCLC. Relative mRNA expression of SIX2 (**b**), SIX3 (**d**), and SIX4 (**f**) between III–IV and I–II patients in ADC

Three SIX genes among the SIX family, namely SIX2, SIX3, and SIX4, were remarkably correlated with the TNM stage of NSCLC. The increased expressions of these genes were associated with advanced tumor stage (SIX2: pooled OR = 1.30, 95 % CI, 1.00–1.68, *p* = 0.467, and *I* ^2^ = 0.0 %; SIX3: pooled OR = 1.56, 95 % CI, 1.20–2.04, *p* = 0.664, and *I* ^2^ = 0.0 %; SIX4: pooled OR = 1.95, 95 % CI, 1.36–2.79, *p* = 0.159, and *I* ^2^ = 39.3 %; Fig. 4a, c, e). Moreover, the same analysis was also conducted on ADC. The relationship between SIX2 expression and TNM stage of ADC was on the verge of statistically significant (pooled OR = 1.39, 95 % CI, 0.95–2.02, *p* = 0.359, and *I* ^2^ = 8.3 %; Fig. 4b). However, SIX3 and SIX4 gave specific tendency in our study (SIX3: pooled OR = 1.81, 95 % CI, 1.22–2.67, *p* = 0.513, and *I* ^2^ = 0.0 %; SIX4: pooled OR = 1.80, 95 % CI, 1.18–2.77, *p* = 0.336, and *I* ^2^ = 11.3 %; Fig. 4d, f). Subgroup analysis of lung squamous cell carcinoma (SQC) showed no significance in SIX2 and SIX3 (see Additional file 1). Among included databases in our study, GSE68793 (Meyerson M. 2015) with 135 SQC patients was derived from TCGA database. The ORs of SIX2 and SIX3 between III–IV and I–II in GSE68793 (SIX2: OR = 1.62; SIX3: OR = 1.62) showed consistent trend with pooled OR.

### The SIX family gene expressions correlated with LNM of NSCLC

The correlation between SIX3 expression and LNM of NSCLC hovered around significance (pooled OR = 1.15, 95 % CI, 0.95–1.39, *p* = 0.050, and *I* ^2^ = 57.7 %; see Additional file 2). The high SIX4 and SIX6 expressions were linked with the greater possibility of LNM in NSCLC (SIX4: pooled OR = 3.07, 95 % CI, 1.60–5.92, *p* = 0.944, and *I* ^2^ = 0.0 %; SIX6: pooled OR = 1.22, 95 % CI, 1.00–1.48, *p* = 0.429, and *I* ^2^ = 0.0 %; see Additional file 2). Other members of the SIX family showed no association with LNM (see Additional file 2).

### The different expressions of the SIX family genes between lung adenocarcinoma and squamous carcinoma

**Fig. 5:**
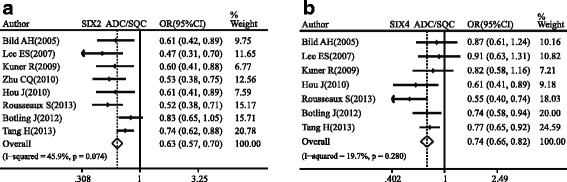
Forest plot of odds ratio (OR). *CI* confidence interval. Relative expression of SIX2 (**a**) and SIX4 (**b**) in ADC compared to SQC

The relative expressions of SIX2 and SIX4 were found higher in SQC compared with ADC in NSCLC (SIX2: pooled OR = 0.63, 95 % CI, 0.57–0.70, *p* = 0.074, and *I* ^2^ = 45.9 %; SIX4: pooled OR = 0.74, 95 % CI, 0.66–0.82, *p* = 0.280, and *I* ^2^ = 19.7 %; Fig. 5a, b).

### Impact of the SIX family expression on overall survival for NSCLC

**Fig. 6:**
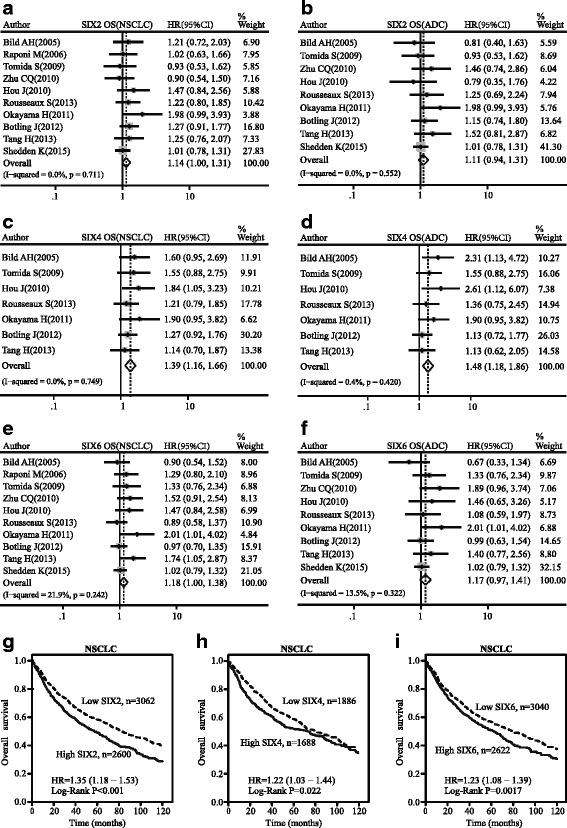
Forest map of hazard ratio (HR) and Kaplan-Meier survival curve. *CI* confidence interval. Association between SIX2 (**a**), SIX4 (**c**), and SIX6 (**e**) with OS of NSCLC. Association between SIX2 (**b**), SIX4 (**d**), and SIX6 (**f**) with OS of ADC. Kaplan-Meier survival curves of SIX2 (**g**), SIX4 (**h**), and SIX6 (**i**) with OS of NSCLS

The prognostic value of the SIX family in NSCLC was analyzed. SIX2, SIX4, and SIX6 showed significant correlation with the poor OS of NSCLC (SIX2: pooled HR = 1.14, 95 % CI, 1.00–1.31, *p* = 0.711, and *I* ^2^ = 0.0 %; SIX4: pooled HR = 1.39, 95 % CI, 1.16–1.66, *p* = 0.749, and *I* ^2^ = 0.0 %; SIX6: pooled HR = 1.18, 95 % CI, 1.00–1.38, *p* = 0.242, and *I* ^2^ = 21.9 %; Fig. 6a, c, e). However, only SIX4 reached statistically significance in poor OS of ADC (pooled HR = 1.48, 95 % CI, 1.18–1.86, *p* = 0.420, and *I* ^2^ = 0.4 %; Fig. 6d). SIX2 and SIX6 approached conventional significance levels (SIX2: pooled HR = 1.11, 95 % CI, 0.94–1.31, *p* = 0.552, and *I* ^2^ = 0.0 %; SIX6: pooled HR = 1.17, 95 % CI, 0.97–1.41, *p* = 0.322, and *I* ^2^ = 13.5 %; Fig. 6b, f). However, SIX2, SIX4, and SIX6 had no significance in improving OS of SQC (see Additional file 3). There was an obvious relationship between SIX5 expression and poor OS rate in SQC, while SIX3 showed positive relation to OS, suggesting a protective effect (see Additional file 4). The Kaplan-Meier curves indicated that patients with higher mRNA levels of SIX2, SIX4, and SIX6 had unfavorable OS time, which represent poor survival in NSCLC (SIX2: pooled HR = 1.35, 95 % CI, 1.18–1.53, *p* < 0.001; SIX4: pooled HR = 1.22, 95 % CI, 1.03–1.44, *p* = 0.022; SIX6: pooled HR = 1.23, 95 % CI, 1.08–1.39, *p* = 0.0017; Fig. 6g–i).

### Effect of the SIX family expression on relapse-free survival for NSCLC

**Fig. 7:**
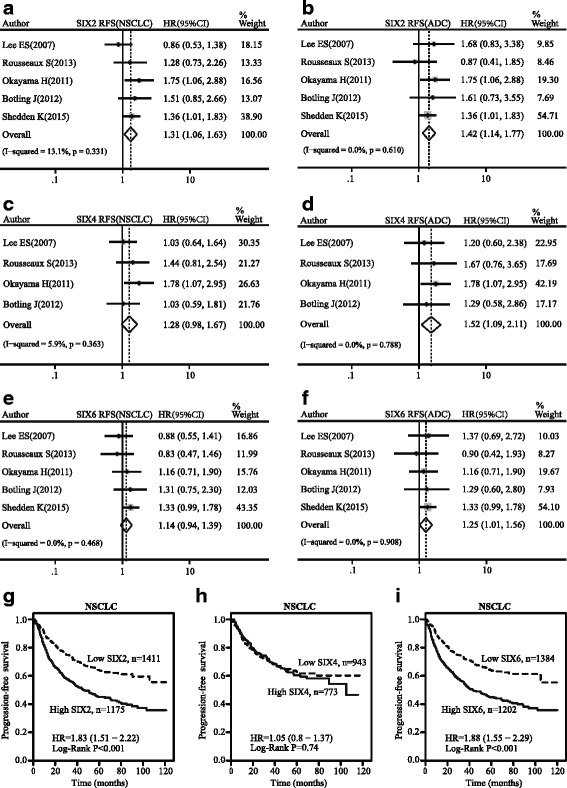
Forest map of hazard ratio (HR) and Kaplan-Meier survival curve. *CI* confidence interval. Association between SIX2 (**a**), SIX4 (**c**), and SIX6 (**e**) with RFS of NSCLC. Association between SIX2 (**b**), SIX4 (**d**), and SIX6 (**f**) with RFS of ADC. Kaplan-Meier survival curves of SIX2 (**g**), SIX4 (**h**), and SIX6 (**i**) with RFS of NSCLS

SIX2 expression was associated with poor RFS in NSCLC (pooled HR = 1.31, 95 % CI, 1.06–1.63, *p* = 0.331, and *I* ^2^ = 13.1 %; Fig. 7a) as well as in ADC (pooled HR = 1.42, 95 % CI, 1.14–1.77, *p* = 0.610, and *I* ^2^ = 0.0 %; Fig. 7b). SIX4 and SIX6 expressions approached statistical significance on poor RFS in NSCLC (SIX4: pooled HR = 1.28, 95 % CI, 0.98–1.67, *p* = 0.363, and *I* ^2^ = 5.9 %; SIX6: pooled HR = 1.14, 95 % CI, 0.94–1.39, *p* = 0.468, and *I* ^2^ = 0.0 %; Fig. 7c, e). Nevertheless, subgroup analysis showed that SIX4 and SIX6 expressions also played a critical role in poor RFS of ADC (SIX4: pooled HR = 1.52, 95 % CI, 1.09–2.11, *p* = 0.788, and *I* ^2^ = 0.0 %; SIX6: pooled HR = 1.25, 95 % CI, 1.01–1.56, *p* = 0.908, and *I* ^2^ = 0.0 %; Fig. 7d, f). SIX3 had a positive effect on RFS of SQC (see Additional file 4), but other SIX members did not reach a statistical significance (see Additional files 4 and 5). The Kaplan-Meier curves showed that SIX2 and SIX6 expressions predicted poor RFS of patients diagnosed as NSCLC (SIX2: pooled HR = 1.83, 95 % CI, 1.51–2.22, *p* < 0.001; SIX6: pooled HR = 1.88, 95 % CI, 1.55–2.29, *p* < 0.001; Fig. 7g, i), while SIX4 had no significant effect (pooled HR = 1.05, 95 % CI, 0.8–1.37, *p* = 0.74; Fig. 7h).

### Publication bias

Begg’s test and Egger’s test were employed to get publication bias statistics. And it did not indicate significant publication bias for the following parameters. SIX1 mRNA expression: NSCLC/normal: Begg’s test *p* = 0.707, Egger’s test *p* = 0.901; ADC/normal: Begg’s test *p* = 0.806, Egger’s test *p* = 0.604. SIX2 mRNA expression: NSCLC/normal: Begg’s test *p* = 1.000, Egger’s test *p* = 0.808; ADC/normal: Begg’s test *p* = 0.806, Egger’s test *p* = 0.577; III~IV/I~II: Begg’s test *p* = 0.386, Egger’s test *p* = 0.324; ADC-III~IV/ADC-I~II: Begg’s test *p* = 0.806, Egger’s test *p* = 0.405; OS of NSCLC: Begg’s test *p* = 0.371, Egger’s test *p* = 0.238; OS of ADC: Begg’s test *p* = 0.466, Egger’s test *p* = 0.415; RFS of NSCLC: Begg’s test *p* = 0.806, Egger’s test *p* = 0.980; RFS of ADC: Begg’s test *p* = 0.806, Egger’s test *p* = 0.996. SIX3 mRNA expression: NSCLC/normal: Begg’s test *p* = 1.000, Egger’s test *p* = 0.620; ADC/normal: Begg’s test *p* = 0.806, Egger’s test *p* = 0.904; III~IV/I~II: Begg’s test *p* = 0.536, Egger’s test *p* = 0.255; ADC-III~IV/ADC-I~II: Begg’s test *p* = 0.806, Egger’s test *p* = 0.479. SIX4 mRNA expression: NSCLC/normal: Begg’s test *p* = 0.734, Egger’s test *p* = 0.656; ADC/normal: Begg’s test *p* = 1.000, Egger’s test *p* = 0.702; III~IV/I~II: Begg’s test *p* = 0.086, Egger’s test *p* = 0.070; ADC-III~IV/ADC-I~II: Begg’s test *p* = 0.308, Egger’s test *p* = 0.165; ADC/SQC: Begg’s test *p* = 0.548, Egger’s test *p* = 0.871; OS of NSCLC: Begg’s test *p* = 0.230, Egger’s test *p* = 0.062; RFS of NSCLC: Begg’s test *p* = 0.734, Egger’s test *p* = 0.871; RFS of ADC: Begg’s test *p* = 0.734, Egger’s test *p* = 0.381. SIX5 mRNA expression: NSCLC/normal: Begg’s test *p* = 1.000, Egger’s test *p* = 0.638; ADC/normal: Begg’s test *p* = 0.086, Egger’s test *p* = 0.149. SIX6 mRNA expression: OS of ADC: Begg’s test *p* = 0.251, Egger’s test *p* = 0.196; RFS of NSCLC: Begg’s test *p* = 0.806, Egger’s test *p* = 0.261; RFS of ADC: Begg’s test *p* = 0.462, Egger’s test *p* = 0.377.

## Discussion

Molecular classification and targeted therapy improve outcomes of NSCLC. For example, novel agents targeting epidermal growth factor receptor (EGFR) mutation and echinoderm microtubule-associated protein-like anaplastic lymphoma kinase (EML4-ALK) fusion benefit the patients with advanced ADC [22, 23]. Other molecular alterations, such as Notch signaling, NLK, and NRF2, also contribute to NSCLC progression [24–26]. Accordingly, exploring new molecular markers is conducive to precision treatment. RDGN is a crucial signal of organ development, especially in lung tissue [1–5]. Aberrant expression of RDGN confers to tumorigenesis [27, 28]. SIX and EYA were considered as components of RDGN, which coordinationally regulated cell proliferation, apoptosis, tumor growth, and metastasis [27, 28]. Recent genetic study indicated that another RDGN member DACH1 is a promising tumor suppressor [29] and mechanism analysis demonstrated that DACH1 inhibited cancer proliferation and invasion [30–32]. Consequently, the detection of this pathway might be used to monitor tumor progression and predict prognosis of cancer patients.

It has been acknowledged that the SIX family correlated with progression and prognosis of a diverse range of tumors [27, 28, 33]. Among them, the SIX1 has been extensively studied [34]. It has been reported that SIX1 showed great influence on cell proliferation, survival, and motility by transcriptional regulating cyclin D1 and c-myc in rhabdomyosarcoma [35] and cyclin A1 in breast cancer in vitro [36]. Thus, inappropriate expression of SIX1 can both induce tumorigenesis and promote metastasis [37]. Nevertheless, the role of SIX1 in NSCLC has not been eastablished. In this study, we found that mRNA level of SIX1 was higher in whole NSCLC tissues compared with normal tissues as well as in ADC, indicating that SIX1 might be involved in the tumorigenesis of NSCLC. Xia Y et al. reported that the expression of SIX1 was associated with heavy tumor burden, including large tumor size, advanced tumor stage, and distant metastasis of NSCLC [15]. In in vitro study, they demonstrated that silencing of endogenous SIX1 attenuated proliferation and invasion of lung cancer, which supported our clinical analysis [15]. But the role of SIX1 in relation to other clinicopathological parameters, such as TNM stage and LNM, was ambiguous in our analysis. We also found that there was no definite predictive value of SIX1 for the prognosis of NSCLC. However, Mimae T et al. reported that in microdesected tissue, mRNA and protein expressions of SIX1 were elevated in minimally invasive ADC and double upregulation of Notch2 and SIX1 contributed to preinvasive-to-invasive transition in ADC, suggesting that SIX1 contributed to the progression of ADC [16]. Possible explanation is that no-tumor cell contamination in no-microdesected tumor tissue may attenuate the real expression level of SIX1 in cancer. In addition, increased SIX1 protein abundance might derive from enhanced translational regulation and protein stability through modification. Anyway, functional activation of SIX1 may promote tumor progression.

Our study also demonstrated that the mRNA level of SIX2 was higher in NSCLC tissues than in normal tissues, suggesting that SIX2 might participate in the tumorigenesis of NSCLC. Furthermore, we found that high SIX2 expression was positively correlated with the high stage which exhibits a greater possibility of invasiveness and poor prognosis in NSCLC. The OS and RFS time were shorter in NSCLC patients with higher expression of SIX2, whereas subgroup analysis did not reach significance in ADC and SQC. The mouse model study has proposed that Six2 promoted breast cancer metastasis by reducing the E-cadherin [38]. This may explain the unfavorable prognosis associated with SIX2 in NSCLC.

SIX3, unlike other members of the SIX family, is a suppressor in proliferation and migration of lung cancer cells. Min-Li Mo et al. demonstrated for the first time that SIX3 over-expression repressed a number of oncogenic genes related to proliferation and metastasis during lung carcinogenesis [18]. Furthermore, SIX3 expression was identified to be associated with improved RFS and OS in the early stage of ADC patients [18]. Considering SIX3 is the only SIX family member that does not interact with EYA [11, 13], which is an oncogene, it is reasonable to comprehend the tumor suppression features of SIX3. Although there was no-statistic significance between SIX3 expression and the OS as well as RFS of NSCLC patients at the mRNA level in our analysis, subgroup analysis still suggested that SIX3 could become a prognostic marker for SQC. Paradoxically, we found that the high expression of SIX3 was detected in tumor tissues and it was related to the advanced stage in NSCLC or ADC but not in SQC. Based on our result and previous publication, we proposed that SIX3 might promote the initiation of NSCLC, but inhibit tumor progression once tumor formed, especially in the early stage of lung squamous cell carcinoma.

Another intriguing finding of our analysis is the expression of SIX4 in NSCLC, which has been rarely studied. The SIX4 expression was higher in NSCLC or ADC than in normal tissues. Detailed analysis suggested a higher expression of SIX4 in SQC than ADC. Significant correlation between SIX4 expression and high TNM stage of NSCLC or ADC was found at mRNA level. But insufficient researches among our inclusive literatures referred to the association between SIX4 expression and TNM stage of SQC. Higher expression of SIX4 conferred to the greater possibility of LNM. Moreover, patients with higher SIX4 expression showed significantly poor OS and RFS in NSCLC or ADC. As mentioned above, SIX4 has the potential to be a novel biomarker in screening out high risk patients and assessing prognosis of NSCLC.

SIX5 was supposed as an epithelial differentiation marker in ovary tissue [39]_._ There was no published research about the correlation between SIX5 and NSCLC. Our meta-analysis indicated that the SIX5 was involved in tumor formation of NSCLC. Apparently, higher expression of SIX5 was associated with poor OS in SQC. Therefore, SIX5 may predict the prognosis of SQC.

High frequency methylation of SIX6 promoter was detected in early stage of NSCLC [17]. Although those methylations were associated with clinical characteristics of NSCLC, the biological functions were not addressed. Our analysis revealed that SIX6 was associated with a greater possibility of LNM in NSCLC. Consistently, SIX6 were linked to the poor OS in NSCLC and poor RFS in ADC. Based on the hypothesis that SIX6 regulated proliferation by directly repressing anti-oncogene p27^Kip1^ during mammalian retinogenesis and pituitary development [40], we inferred that inappropriate activation of SIX6 may promote proliferation of NSCLC by promoting cell cycle progress.

Heterogeneity tests are indispensable for a meta-analysis. In this analysis, minor heterogeneities were observed. There are several reasons for the heterogeneities, including (1) whole genomic expression profiles provide unbiased quantitative measure of mRNA expression; however, different platforms of gene expression array might produce the heterogeneity. (2) Confounding factors such as race and histology also augment heterogeneities in our analysis. (3) Multicenter prospective studies based on large sample size are required.

Publication bias analysis of clinicopathological parameter and survival showed no big variation. In addition, the following limitations should be considered in our analysis: (1) we cannot eliminate the potential publication bias; (2) the inclusive literatures were limited; (3) the methods for detecting SIX gene expression and cut-off values were different; and (4) raw data of some researches were not available when conducting this meta-analysis.

## Conclusions

In conclusion, the meta-analysis revealed that the SIX family might play a pivotal role in the initiation and progression of NSCLC, especially in ADC. Each member of SIX family has its own characteristic in tumorigenesis of NSCLC. SIX1, SIX2, SIX3, SIX4, and SIX5 were detected at high expression levels in NSCLC tissues. SIX2, SIX3, and SIX4 were linked to high TNM stages. Higher expressions of SIX4 and SIX6 correlated with the greater possibility of LNM. Moreover, the expressions of SIX2, SIX4, and SIX6 in NSCLC or in ADC indicated poor OS and RFS. SIX3 and SIX5 were related to OS and RFS of SQC. Overall, the measurement of the SIX family provides potential approaches to molecular diagnosis, evaluation of prognosis, and targeted therapy of NSCLC in the future.

## Additional files

Additional file 1: Relative mRNA expression of SIX2 and SIX3 between III-IV and I-II patients in SQC. (PDF 403 kb)
Additional file 2: Association between mRNA expression of SIX family and NSCLC lymph node metastasis. (PDF 456 kb)
Additional file 3: Association between SIX2, SIX4, SIX6 and OS in SQC. (PDF 386 kb)
Additional file 4: Association between SIX3, SIX5 and OS, RFS in SQC. (PDF 418 kb)
Additional file 5: Association between SIX2, SIX4, SIX6 and RFS in SQC. (PDF 382 kb)

## Abbreviations

ADC: Lung adenocarcinoma
CI: Confidence interval
EGFR: Epidermal growth factor receptor
EML4-ALK: Echinoderm microtubule-associated protein-like anaplastic lymphoma kinase
EMT: Epithelial-mesenchymal transition
HD: Homeobox nucleic acid recognition domain
HR: Hazard ratio
LNM: Lymph node metastasis
NOS: Newcastle-Ottawa Quality Assessment Scale
NSCLC: Non-small cell lung cancer
OS: Overall survival
PFS: Progression-free survival
PH: Pulmonary hypoplasia
RCTs: Randomized controlled studies
RDGN: Retinal Determination Gene Network
RFS: Relapse-free survival
SD: SIX domain
SQC: Lung squamous cell carcinoma
