# Supplementary material for: The expression profile and clinic significance of the SIX family in non-small cell lung cancer
Source: J Hematol Oncol. 2016 Nov 8;9:119. doi: 10.1186/s13045-016-0339-1 (PMC5100270; doi:10.1186/s13045-016-0339-1)
Supplement: Additional file 1: — Relative mRNA expression of SIX2 and SIX3 between III-IV and I-II patients in SQC. (PDF 403 kb) [file 13045_2016_339_MOESM1_ESM.pdf]

The expression profile and clinic significance of the SIX family in non-small cell lung cancer

Qian Liu[1], Anping Li[2], Yijun Tian[1], Yu Liu[3], Tengfei Li[2], Cuntai Zhang[3], Jennifer D. Wu[4], Xinwei Han[2], Kongming Wu[1]

1. Department of Oncology, Tongji Hospital of Tongji Medical College, Huazhong University of Science and Technology, 1095 Jiefang Avenue, Wuhan 430030, P.R. China.
2. Department of Interventional Radiology, The First Affiliated Hospital of Zhengzhou University, Zhengzhou, 450052, China.
3. Department of Geriatric, Tongji Hospital of Tongji Medical College, Huazhong University of Science and Technology, Wuhan, 430030, China.
4. Department of Microbiology and Immunology, Hollings Cancer Center, Medical University of South Carolina, Charleston, South Carolina, USA

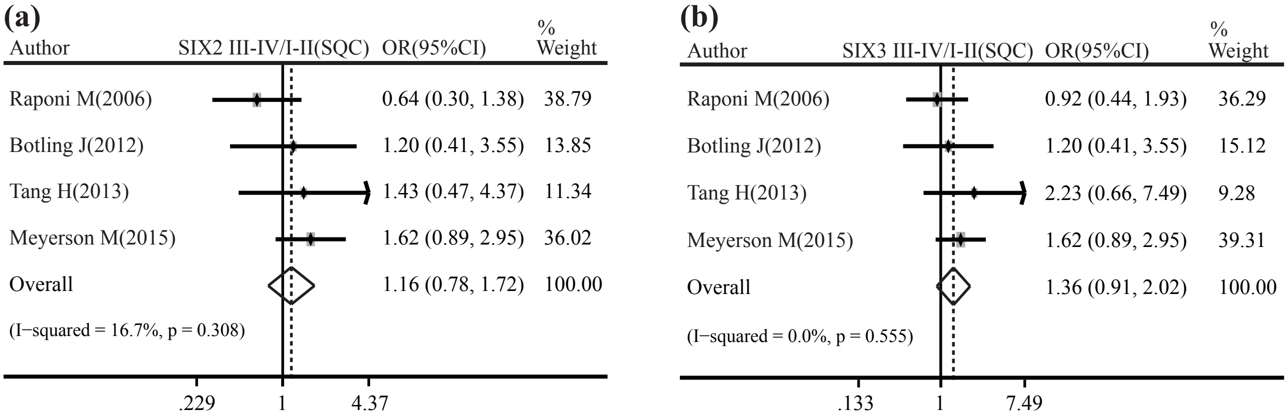

Additional file 1. Forest plot of odds ratio (OR). CI, confidence interval. **(a).** Relative mRNA expression of SIX2 between III-IV and I-II patients in SQC. **(b).** Relative mRNA expression of SIX2 between III-IV and I-II patients in SQC.
