# Supplementary material for: The expression profile and clinic significance of the SIX family in non-small cell lung cancer
Source: J Hematol Oncol. 2016 Nov 8;9:119. doi: 10.1186/s13045-016-0339-1 (PMC5100270; doi:10.1186/s13045-016-0339-1)
Supplement: Additional file 2: — Association between mRNA expression of SIX family and NSCLC lymph node metastasis. (PDF 456 kb) [file 13045_2016_339_MOESM2_ESM.pdf]

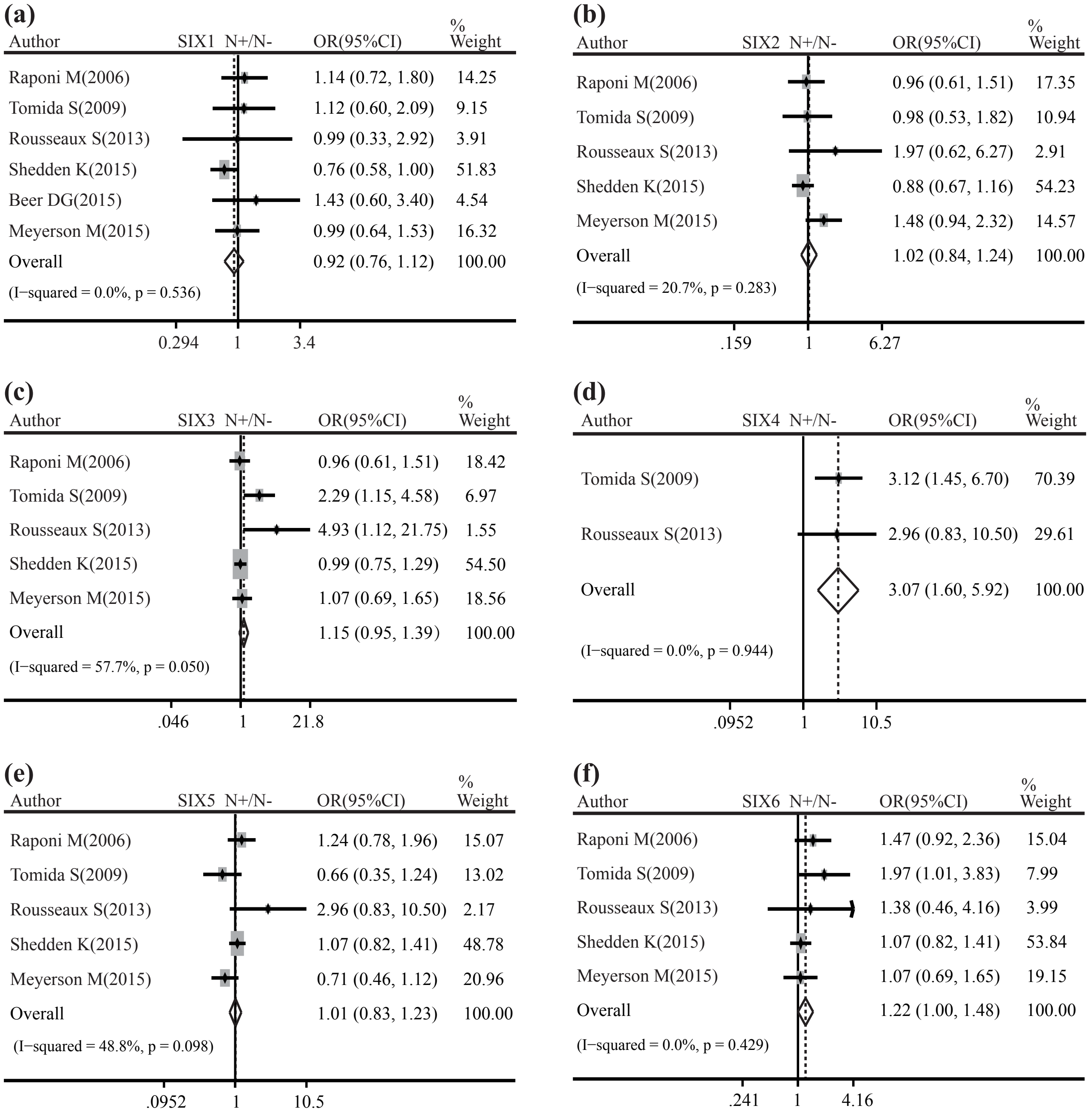

Additional file 2. Forest plot of odds ratio (OR). CI, confidence interval. **(a).** Association between mRNA expression of SIX1 and NSCLC lymph node metastasis. **(b).** Association between mRNA expression of SIX2 and NSCLC lymph node metastasis. **(c).** Association between mRNA expression of SIX3 and NSCLC lymph node metastasis. **(d).** Association between mRNA expression of SIX4 and NSCLC lymph node metastasis. **(e).** Association between mRNA expression of SIX5 and NSCLC lymph node metastasis. **(f).** Association between mRNA expression of SIX6 and NSCLC lymph node metastasis.
