# Supplementary material for: The expression profile and clinic significance of the SIX family in non-small cell lung cancer
Source: J Hematol Oncol. 2016 Nov 8;9:119. doi: 10.1186/s13045-016-0339-1 (PMC5100270; doi:10.1186/s13045-016-0339-1)
Supplement: Additional file 4: — Association between SIX3, SIX5 and OS, RFS in SQC. (PDF 418 kb) [file 13045_2016_339_MOESM4_ESM.pdf]

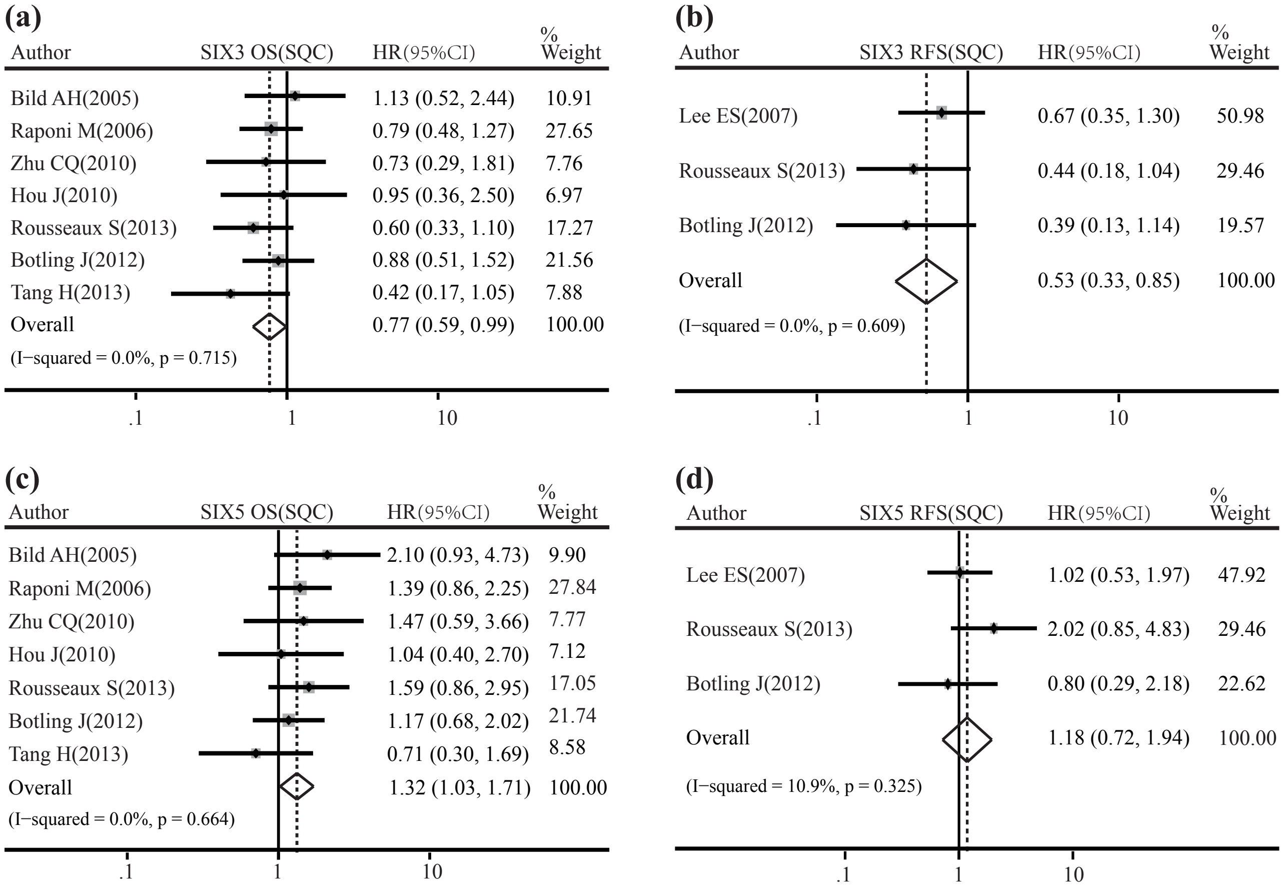

Additional file 4. Forest map of hazard ratio(HR). CI, confidence interval. **(a).** Association between SIX3 and SQC OS. **(b).** Association between SIX3 and SQC RFS. **(c).** Association between SIX5 and SQC OS. **(d).** Association between SIX5 and SQC RFS.
