# Supplementary material for: The expression profile and clinic significance of the SIX family in non-small cell lung cancer
Source: J Hematol Oncol. 2016 Nov 8;9:119. doi: 10.1186/s13045-016-0339-1 (PMC5100270; doi:10.1186/s13045-016-0339-1)
Supplement: Additional file 5: — Association between SIX2, SIX4, SIX6 and RFS in SQC. (PDF 382 kb) [file 13045_2016_339_MOESM5_ESM.pdf]

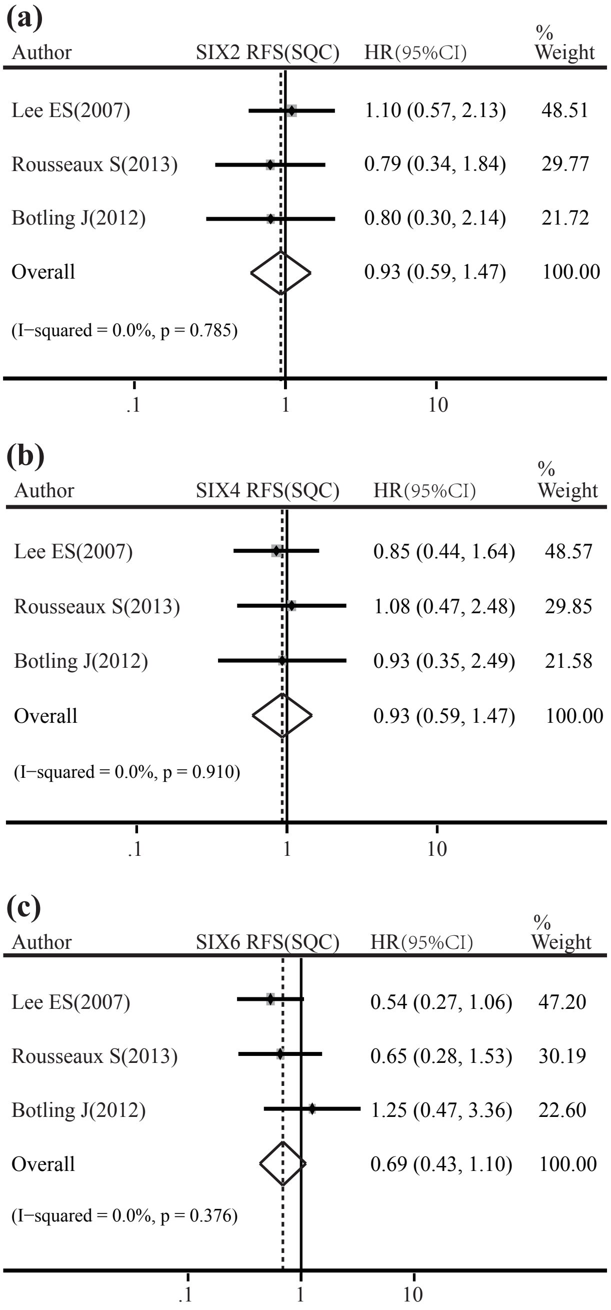

Additional file 5. Forest map of hazard ratio(HR). CI, confidence interval. **(a).** Association between SIX2 and SQC RFS. **(b).** Association between SIX4 and SQC RFS. **(c).** Association between SIX6 and SQC RFS.
